# 

**Published:** 1825-07-01

**Authors:** 


### Contents

OF

No. 5. Vol. III. (NEW SERIES) for JULY, 1825.

[*No.* 21, *ANALYTICAL SERIES.*]

Art. I.

An Account of the Disease lately prevalent at the General Penitentiary. By P. Mere Latham M.D. .. .. .. .. 1

II.

Mr. Charles Bell on Injuries of the Spine and of the Thighbone .. .. .. .. .. .. .. .. .. .. .. 41

III.

A Fasciculus of Nine Lithographic Anatomical Drawings, from Preparations in the Museum of the Army Medical Department at Chatham .. .. .. .. .. .. .. .. .. 57

IV

Elements of the Etiology and Philosophy of Epidemics. By Joseph Mather Smith, M.D. of New York .. .. .. 62

V.

Dr. Venables' Clinical Report on Dropsies; with an Appendix on the Theory and Treatment of Organic Disease in general 88

VI.

Dr. Ratier on the Hospital Practice of Paris .. .. .. .. 94

VII.

Mr. Buchanan's Illustrations of Acoustic Surgery .. .. .. 111

VIII.

Report of the Epidemic Cholera, as it appeared in the Territories subject to the Presidency of Fort St. George. By William Scot, Surgeon, and Secretary to the Medical Board .. 121

IX.

Mr. Brande's Manual of Pharmacy .. .. .. .. .. .. 141

X.

Analysis of Medical Evidence, comprising Directions for Practitioners, as Witnesses in Courts of Justice. By JOHN GORDON SMITH, M.D. .. .. .. .. .. .. .. .. .. 169

XI.

Elements of Pathology and Therapeutics, &c. By CALEB HILLIER PARRY, M.D. Vol. I. *Second Edition.* With Appendix of New Matter. .. .. .. .. .. .. .. .. 188

XII.

### Quarterly Periscope

OF

### PRACTICAL MEDICINE;

BEING

*THE SPIRIT OF THE MEDICAL JOURNALS,*

FOREIGN AND DOMESTIC.

I. PHYSIOLOGY.

1. Vivisections—Consistency of the Medical Saints .. .. .. 198
2. Messrs. Breschet and Edwards' Experiments to ascertain the Influence of the Pneumo-gastric Nerves on Digestion.. .. 200
3. Dr. Wollaston on the Semi-decussation of the Optic Nerves.. 201
Vicq. D'Azyr, the Wenzels, and Treveranus, on the same Subject. .. .. .. .. .. .. .. .. .. .. .. .. 203
4. M. Billard's Cases of Partial Paralysis of the Face, from local Pressure on the Portio Dura of the Auditory Nerve .. .. 203
Dr. Johnson's Case of the same kind .. .. .. .. .. 205

II. PATHOLOGY.

1. M. Bayle on Angina Œdematosa, with Cases .. .. .. 205
Dr. Beck, of New York, on the same subject. .. .. .. 208
The Editor's Case of Congestive Tracheitis saved by extreme Bleeding .. .. .. .. .. .. .. .. .. .. .. 209
2. Case of Fatal Irritative Fever, by Mr. Anderson.. .. .. 211
3. Dr. Duncan on Inflammation of the Cellular Membrane .. 212
4. Acute Cephalitis simulating Hepatitis .. .. .. .. .. 216
5. Pulmonic Disease, by Dr. Cobb .. .. .. .. .. .. .. 217
6. Professor Recamier's Hospital Reports.. .. .. .. .. 217
A. Case of Fatal Remittent Fever. .. .. .. .. .. 218
B. Paracentesis Thoracis, with prospect of recovery .. .. 219
C. Curious and Fatal Case of Dyspnœa .. .. .. .. 219
D. Perforation of the Stomach .. .. .. .. .. .. 220

Successful Puncture of an Encysted Tumour in the Hypochondrium.. .. .. .. .. .. .. .. .. .. 221
7. Fatal case of Cystitis Hepatica .. .. .. .. .. .. 222
8. M. Menard on the Seat and Cause of Epilepsy .. .. .. 223
9. Mr. Dundas on Concussion of the Spine .. .. .. .. 225
10. M. Velpeau on Hæmorrhagic and Cerebriform Tumours .. 226
11. Dr. Coventry on the Cause of Bronchocele .. .. .. .. 228
12. M. Neuman on the Pathology of Insanity .. .. .. .. 231
13. M. Velpeau on the Pathology of Phlegmatia Dolens .. .. 233
14. Cases of Obliteration of the Biliary Ducts .. .. .. .. 237
15. On Suppression of Urine .. .. .. .. .. .. .. .. 240
16. Hæmatemesis fatal in 14 Hours .. .. .. .. .. .. .. 240
17. M. Bricheteau on Cardiac Aneurism in Young People . .. 241
18. Pericarditis and other Phlegmasiæ from a Moral Cause. .. 242

III. SURGERY.

1. Professor Delpech's Case of Wounded Carotid Artery .. .. 244
2. Mr. Liston on Lithotomy. .. .. .. .. .. .. .. .. 247
3. Mr. Shaw on Wounds received in Dissection.. .. .. .. 249
4. Dr. Thomson's Case of Poisoned Wound in his own Person.. 253
5. M. Pelletan on Acupuncture .. .. .. .. .. .. .. .. 257
M. Meyrans on ditto. .. .. .. .. .. .. .. .. .. 260
6. On Extirpation of the Uterus .. .. .. .. .. .. .. .. 264
7. On Partial Amputation of the Hand . .. .. .. .. .. 267
8. Baron Larrey on a New Method of Treating Compound Fractures .. .. .. .. .. .. .. .. .. .. .. .. 269

IV. THERAPŒIA.

1. On Acute Dysentery . .. .. .. .. .. .. .. .. .. 270
2. Intermittent Affection of the Chest.. .. .. .. .. .. 272
3. On the Oil of Euphorbia Lathyrus, a Substitute for Croton .. 273
4. Extraction of Poison from the Stomach .. .. .. .. .. .. 276
5. On Iodine in Syphilitic Affections .. .. .. .. .. .. 277
6. Mr. Davis on Meningitis . .. .. .. .. .. .. .. .. 279
7. On Partial Paralysis of the Face .. .. .. .. .. .. 281
8. Puncture—Tetanus—Stimulants .. .. .. .. .. .. 283
9. Dr. Belcher on Purpura Hæmorrhagica.. .. .. .. .. 284

V. MIDWIFERY.

1. Cæsarean Operations. .. .. .. .. .. .. .. .. .. 285
2. Uterine Tumour .. .. .. .. .. .. .. .. .. .. 286
3. Rupture of Perineum .. .. .. .. .. .. .. .. .. 287

VI. MEDICAL JURISPRUDENCE.

1. Messrs. Orfila and Vauquelin on a Charge of Poisoning by Arsenic.. .. .. .. .. .. .. .. .. .. .. .. 289

2. Contagion—Plague—Quarantine .. .. .. .. .. .. 292
3. Senatus Academicus Edinensis . .. .. .. .. .. .. .. 294
4. Dr. R. Ferguson on the Policy of Variolo-Vaccination.. .. 295
5. Decision of the Trial—Abernethy *versus* the Lancet—Injunction granted .. .. .. .. .. .. .. .. .. .. .. .. 297

XIII.

ANALECTA MINORA.

1. Nitre in Menorrhagia .. .. .. .. .. .. .. .. 298
2. Iodine in Sulphureous Springs .. .. .. .. .. .. 298
3. Sangrado Practice, revived in France .. .. .. .. .. .. 299
4. Hydrophobia .. .. .. .. .. .. .. .. .. .. 299
5. M. Pinel, jun. on Deafness in Old People .. .. .. .. 299
6. Belladonna externally applied in Tic Douloureux.. .. .. 300
7. On the injection of Dead Bodies with Whiskey .. .. .. 300
8. Dr. Godman on Dissection Wounds, (Preventive Measure) . 300
9. Fatal Effects from Acupuncture .. .. .. .. .. .. .. 300
10. Iron Nail swallowed with Impunity .. .. .. .. .. .. 301
11. Mr. Amesbury on Fractures of the Patella and Olecranon .. 301

XIV.

Bibliographical Record .. .. .. .. .. .. .. .. .. .. 301

XV.

EXTRA-LIMITES.

I. Mr. Snell's Improved Artificial Nose .. .. .. .. .. .. 305
II. Dr. Scott on Acute Hydrocephalus. .. .. .. .. .. 306
III. Report of the Associated Apothecaries. .. .. .. .. .. 309
IV. Medical Officers of the Crown Life Assurance Company .. 310

XVI.

INTELLIGENCE, CORRESPONDENCE, &c.

Medical Science in South America . .. .. .. .. .. .. .. 313

Invalids going to the Continent for the Winter .. .. .. .. 314

General Meeting of Associated Apothecaries. .. .. .. .. 314

Penetrating Wounds of the Chest .. .. .. .. .. .. .. 314

Mr. Weiss's Instruments (see Plate) .. .. .. .. .. .. .. 315

